# Acupuncture at Tiaokou (ST38) for Shoulder Adhesive Capsulitis: What Strengths Does It Have? A Systematic Review and Meta-Analysis of Randomized Controlled Trials

**DOI:** 10.1155/2018/4197659

**Published:** 2018-04-22

**Authors:** Chao Yang, TaoTao Lv, TianYuan Yu, Steven Wong, MengQian Lu, YiZhen Li

**Affiliations:** School of Acupuncture, Moxibustion and Tuina, Beijing University of Chinese Medicine, Beijing, China

## Abstract

**Objective:**

Tiaokou (ST38) is used as a crucial distal acupoint for treating shoulder adhesive capsulitis (SAC) in traditional Chinese medicine. The objective of this study was to assess the effectiveness and safety of acupuncture at Tiaokou for treating SAC.

**Methods:**

We searched eight electronic databases without language restrictions. All the literature was processed to identify RCTs comparing acupuncture at Tiaokou with other therapies (e.g., acupuncture at local shoulder acupoints and nonsteroidal anti-inflammatory drugs). Two reviewers extracted trials and collected outcome data independently. A meta-analysis was performed following a strict methodology.

**Results:**

19 RCTs involving 1944 participants met our inclusion criteria. The majority of the trials were determined to be of low quality. Positive results were found for acupuncture treatment at Tiaokou (as sole treatment or in combination with shoulder acupoints), which resulted in an improved percentage of clinical effectiveness and Constant-Murley Score values, as well as a reduction in Visual Analogue Scale values of SAC patients.

**Conclusion:**

Our review found encouraging evidence for the effectiveness of acupuncture at Tiaokou for SAC. Nonetheless, despite stringent methodological analyses, these results need to be strengthened by additional RCTs of higher quality.

## 1. Introduction

Shoulder adhesive capsulitis (SAC), which is also known as scapulohumeral periarthritis or frozen shoulder, usually refers to a chronic aseptic inflammation caused by injury or degeneration of the shoulder joint and articular capsule [1]. The main symptoms of SAC are chronic shoulder pain and a restricted range of shoulder movements [2]. Epidemiological investigation has revealed that the incidence of SAC is about 2% to 5% in the general adult population and 10% to 20% in diabetics worldwide [3, 4]. SAC is a chronic, progressive, and self-limiting disease. The conventional treatments for SAC are cortisone injections, nonsteroidal anti-inflammatory drugs (NSAIDs), and physiotherapy [5]. Unfortunately, none of these treatments are proven to be clearly effective for SAC in the long-term [6], and the majority of these interventions are often accompanied by varying degrees of side-effects [7].

In traditional Chinese medicine (TCM), SAC belongs to “Bi Syndrome,” which is usually caused by an invasion of wind-cold-dampness or Qi-Blood stagnation [8]. From ancient times to the present day, acupuncture was regularly applied to relieve the symptoms of SAC and Tiaokou (ST38) was commonly used as a crucial distal acupoint. Using elongated needles or electroacupuncture to stimulate Tiaokou can significantly decrease pain and improve the restricted range of shoulder movements [9]. In recent years, a large amount of clinical research has reported the efficacy and safety of acupuncture at Tiaokou for treating SAC. The objective of this systematic review is to assess the effectiveness and safety of acupuncture at Tiaokou for patients with SAC.

Despite the steady accumulation of evidence concerning the therapeutic effectiveness of acupuncture for SAC over the past several decades, the available reports are still contradictory [10, 11]. The previous systematic reviews mainly focused on the overall curative effect of acupuncture for treating SAC, yet no reviews focused on a conclusive evaluation of acupuncture at a single acupoint in treating SAC. Therefore, we conducted this updated systematic review and meta-analysis to critically evaluate the effectiveness of acupuncture at Tiaokou for treating SAC, in order to provide more robust evidence for decision-makers.

## 2. Method

This systematic review and meta-analysis was conducted according to the guidelines set forth in the Cochrane Handbook for Systematic Reviews of Interventions [12].

### 2.1. Inclusion and Exclusion Criteria

Randomized controlled trials (RCTs) that applied acupuncture at Tiaokou as the therapeutic intervention for treating SAC were included.

*The inclusion criteria were as follows*.

1. Types of studies: only RCTs were included in our review. Quasi-RCTs, crossover trails, cluster-randomized trials, and other study designs were excluded. Our review did not impose any limitations on the publication form or language.
2. Types of participants: studies involving participants who met the diagnostic criteria for SAC were included without limitations related to race or gender. Trials that observed the prognosis of SAC complicated by other disorders (e.g., spinal injury or stroke) were excluded in our review.
3. Types of interventions: trials that compared the effectiveness of acupuncture at Tiaokou with western medicine, acupuncture at shoulder acupoints, or other TCM therapies were included. Our review did not set up any limitations as to the type of acupuncture (including traditional manual acupuncture, elongated needle acupuncture, and electroacupuncture)
4. Types of outcome measures: authoritative indicators were considered in our review for the assessment of symptoms and quality of life of SAC patients, as follows: (i) Visual Analogue Scale (VAS); (ii) Constant-Murley Score (CMS); (iii) percentage of clinical effectiveness; (iv) adverse events.

*The exclusion criteria were as follows*.

Studies were excluded if

1. the data from the study was a repeated publication;
2. the study lacked definite diagnostic criteria;
3. the study lacked details of the intervention or basic information relating to the subjects;
4. the selection of acupoints was unclear in the experimental group;
5. the selection of acupoints was not based on TCM syndrome differentiation in the experimental group;
6. the selection of acupoints for the control group contained Tiaokou.

### 2.2. Literature Search

The following eight databases were searched by two reviewers (Chao Yang and TaoTao Lv) independently: PubMed, MEDLINE, Embase, Cochrane library, CNKI, CBM, VIP, and WanFang database. All the databases were searched from their inception to August 15, 2017. No language restriction was applied in the search strategy. The following search strategy was used for PubMed, as well as for the other electronic databases:

(1) randomized controlled trial; (2) controlled clinical trial; (3) randomized; (4) randomly; (5) placebo; (6) trial; (7) 1 or 2–6
(8) shoulder adhesive capsulitis; (9) adhesive capsulitis; (10) scapulohumeral periarthritis; (11) periarthritis of shoulder; (12) periarthritis humeroscapularis; (13) frozen shoulder; (14) 8 or 9–13
(13) Tiaokou; (14) ST38; (15) elongated needling; (16) contralateral collateral needling; (17) opposing needling; (18) acupuncture; (19) 13 or 14–18
(20) 7 and 12 and 19.

### 2.3. Literature Selection

Two reviewers (Chao Yang and MengQian Lu) independently searched and evaluated every study according to the Cochrane Handbook for Systematic Reviews of Interventions on the basis of the following four procedures: (1) eliminating irrelevant literature by reading the titles and abstracts; (2) the third reviewer (TianYuan Yu) organizing the article results using NoteExpress software and eliminating all the duplicates; (3) eliminating the nonconformity in the literature based on the inclusion criteria by perusing the full text; (4) amalgamating all the literatures that report an identical clinical studies.

### 2.4. Data Extraction and Management

Two reviewers (Chao Yang and TaoTao Lv) independently used a data extraction form to extract data on participants, randomization, interventions, outcomes, duration, follow-up, reason for discontinuation, numbers of treatment-related adverse events, author information, and conflicts of interest. Ethical approval was not needed, because the data did not include individual patient data and therefore no privacy issues would occur. If needed, primary authors of the trials were contacted via email to supplement incomplete data. Furthermore, the “data extraction” form was used for recording and calculating the relevant data of the outcomes.

### 2.5. Assessment of Risk of Bias in Included Studies

Two reviewers (Chao Yang and TaoTao Lv) independently evaluated the risk of bias of the eligible studies using the Cochrane Collaboration's tool. According to the Cochrane risk of bias standards, each study was evaluated for its validity on the following seven aspects: random sequence generation, allocation concealment, blinding of participants and personnel, blinding of outcome assessment, incomplete data assessment, selective outcome reporting, and other sources of bias.

### 2.6. Statistical Analysis

Review Manager 5.4 software was utilized for statistical analysis on the basis of homogeneity of the included trials. Risk ratios (RRs) and 95% confidence intervals (95% CIs) were applied for dichotomous data, for continuous data, mean differences (MDs), and 95% CIs were used for analysis.

To estimate the level of heterogeneity across the studies, we searched for overlapping CI in forest plots and used the *x*^2^ test for statistical heterogeneity and the value of the *I*^2^ statistic (*I*^2^ > 50% or *P* < 0.1 indicating the existence of heterogeneity). A fixed-effect model was applied to calculate the pooled statistics in the absence of substantial heterogeneity (*P* ≥ 0.1 and *I*^2^ < 50%). Conversely, if statistical heterogeneity was identified (*P* < 0.1 or *I*^2^ > 50%), causes of the heterogeneity would be detected first by subgroup analysis and/or sensitive analysis. If the heterogeneity could not readily be explained, a random-effects model would be interpreted with caution.

As more than 10 studies were included in the meta-analysis, funnel plots were used to detect potential reporting biases and small-study effects. The Egger method [32] was used to investigate asymmetry.

## 3. Results

### 3.1. Description of Studies

#### 3.1.1. Literature Search

**Figure 1:**
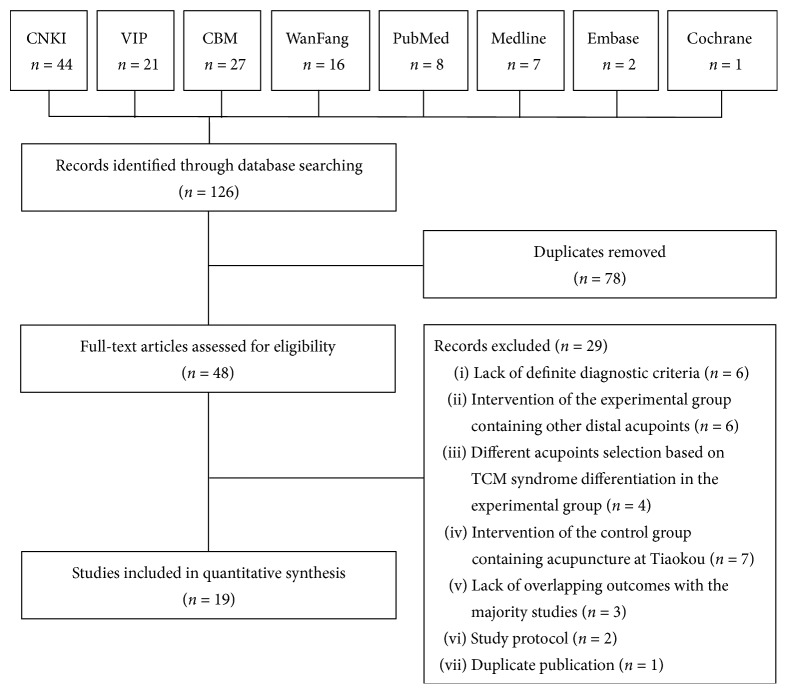
Systematic review process flowchart.

A total of 126 studies were identified by scanning the titles and abstracts. Of these, 78 repetitive studies were eliminated after importing the studies into NoteExpress software, while 48 studies were preliminary included. There were four studies in English and 42 studies in Chinese. In the end, after extensively perusing the full text of the included trials, two articles were study protocol; six studies were eliminated due to a lack of definite diagnostic criteria; 10 studies were eliminated due to the intervention containing other lower limb acupoints or acupoints selection being based on TCM syndrome differentiation in the experimental group; seven studies were eliminated due to the intervention of the control group containing Tiaokou; three studies were eliminated due to lack of overlapping outcomes with the majority of included studies; one study was eliminated due to duplicate publication. In final, there were 19 RCTs that met all the inclusion criteria. All of the included trials were in Chinese, and all were conducted in China. The process of identifying trials is shown in Figure 1.

#### 3.1.2. Study Characteristics

The characteristics of each trial were summarized in Table 1. All of the 19 articles were RCTs published from 2006 to 2016 and performed in China.

*(1) Participants*. The 19 RCTs included a total of 1944 patients (including 770 male, 982 female, and 192 unclear), with sample sizes ranging from 50 to 276. The age of the participants ranged from 35 to 75 years, and all were recruited in China. All of the participants met the diagnostic criteria of SAC. Participants were enrolled as outpatients (or along with inpatients) in hospital setting. All trials limited the age range and medication use of participants.

*(2) Interventions/Controls*. The interventions of the experimental group were contained: (i) penetrating Tiaokou through Chengshan individually [22, 25]; (ii) penetrating Tiaokou through Chengshan in combination with acupuncture at shoulder acupoints [14, 16–21, 26, 27, 29–31]; (iii) penetrating Tiaokou through Chengshan in combination with warm acupuncture at shoulder acupoints [24]; (iv) penetrating Tiaokou through Chengshan in combination with electroacupuncture at shoulder acupoints [23]; (v) electroacupuncture at Tiaokou [13, 15, 28]. All trials required the appearance of postneedling stimulation, known as “De Qi” sensation, a critical factor for the effectiveness of acupuncture therapy. All the experimental interventions in included trials required the participants to demonstrate a small range of motion in the affected shoulder, such as abduction, external rotation, and elevation, when acupuncturists were stimulating Tiaokou. The treatment duration ranged from 5 d to 42 d.

The interventions of control group were contained as follows: (i) prescribing NSAIDs [15, 29]; (ii) acupuncture at shoulder acupoints [13, 16, 18–21, 25, 27, 31]; (iii) electroacupuncture at shoulder acupoints [23, 28]; (iv) warm acupuncture at shoulder acupoints [24]; (v) sham acupuncture at Tiaokou in combination with acupuncture at shoulder acupoints [14, 26, 30]; (vi) Tuina [17]; (vii) gentle range of motion exercises of the affected shoulder [22].

*(3) Outcome Measures*. The percentage of clinical effectiveness was applied as the final outcome in 14 trials [13, 14, 16–24, 29–31]. VAS was measured by 10 trails [13, 15, 17, 21, 22, 25–29] to evaluate the relief of shoulder pain. Four trials [14, 25, 27, 30] evaluated the recovery of shoulder joint movement with CMS.

### 3.2. Methodological Evaluation

**Figure 2:**
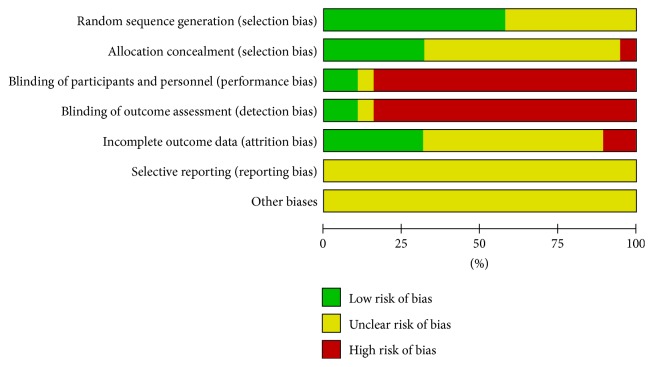
Risk of bias assessment.

**Figure 3:**
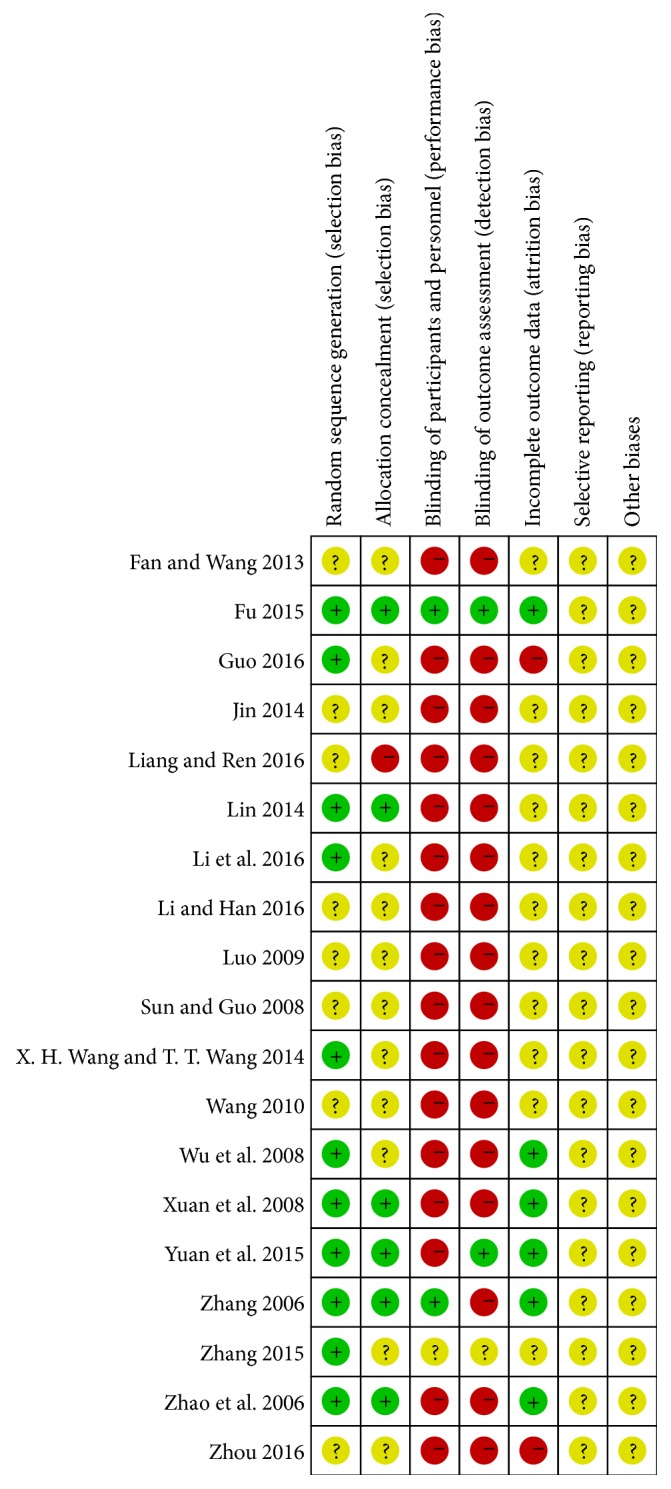
Risk of bias summary.

Results of the risk of bias assessment of the 19 RCTs are presented in Figure 2 and in summary in Figure 3. 11 trials [13–15, 17, 21, 23–26, 28, 31] ensured a defined method of randomization. Furthermore, allocation concealment was considered inadequate in most of the studies, except for six trials [13–15, 23, 25, 26]. Another frequent bias was noted in blinding. Due to the inherent characteristics of acupuncture manipulations, it is hardly possible to conduct blinding, especially for the acupuncturists and participants. Among the included RCTs, only two trials [13, 26] reported the blinding of participants, and two trials [25, 26] blinded the statistical analysts. The presentation of “incomplete outcome data” was lacking in most of the trails, with only six trials [13–15, 17, 25, 26] reporting the details of abscission. None of the included trials reported a selection bias.

### 3.3. Meta-Analysis of the Therapeutic Effects of Acupuncture at Tiaokou

#### 3.3.1. Evaluation of the Percentage of Clinical Effectiveness

In the included RCTs, there were 14 studies [13, 14, 16–24, 29–31] that evaluated the percentage of clinical effectiveness of acupuncture at Tiaokou for treating SAC. Seven studies [18, 19, 21, 23, 24, 29, 31] adopted the criteria of therapeutic effectiveness in the treatment of SAC from* Diagnostic Criteria for Symptoms and Signs of Chinese Medicine* [33], which was formulated by China State Administration of Traditional Chinese Medicine. Three studies [13, 16, 17] adopted the criteria of therapeutic effectiveness found in* Clinical Guidelines of New Drugs for Traditional Chinese Medicine* [34]. One study [22] adopted the criteria of therapeutic effectiveness described in* Clinical Pain Therapeutics* [35]. Three studies [14, 20, 30] formulated self-proposed criteria of therapeutic effectiveness for SAC. Although the standards were inconsistent, all criteria of these studies recorded a relatively unified definition of invalid case: the shoulder pain and restriction of shoulder movement having no obvious improvement. Therefore, in our meta-analysis, we divided the curative effect into effective cases and invalid cases: the number of effective cases = the number of completely healed cases + the number of significantly effective cases + the number of effective cases.

**Figure 4:**
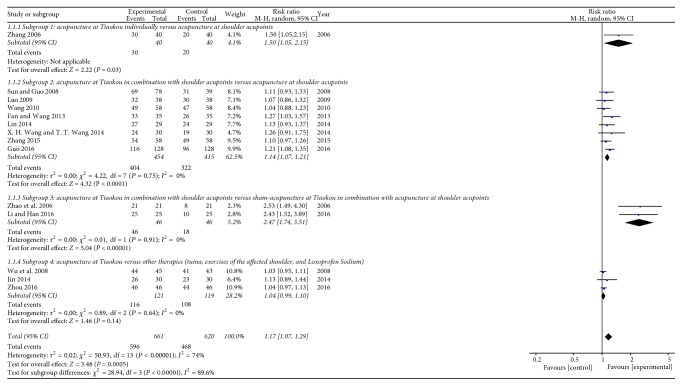
Forest plots of outcome “percentage of clinical effectiveness.”

In this meta-analysis, we extracted and analyzed the data from these 14 studies and the overall effects favored acupuncture at Tiaokou in terms of percentage of clinical effectiveness (RR = 1.17 [1.07, 1.29] (95% CI), *P* = 0.0005; Figure 4). Meanwhile, the heterogeneity test showed a high level of inconsistency across the 14 included studies (*P* < 0.00001; *I*^2^ = 74%; Figure 4). We performed a subgroup analysis to investigate the possible cause of heterogeneity. Even though there was no heterogeneity in these subgroups, the total subgroup differences were significantly substantial (*P* < 0.00001; *I*^2^ = 89.6%; Figure 4). We finally applied a random-effects model.

As the meta-analysis result of subgroup 1 (1 RCT) demonstrated a favorable effect of acupuncture at Tiaokou individually on the percentage of clinical effectiveness (RR = 1.50 [1.05, 2.15] (95% CI), *P* = 0.03; Figure 4, analysis 1.1.1) compared with acupuncture at shoulder acupoints, meta-analysis of subgroup 2 (8 RCTs) showed that the effect of acupuncture at Tiaokou in combination with shoulder acupoints was superior to acupuncture only at local shoulder acupoints (RR = 1.14 [1.07, 1.21] (95% CI) *P* < 0.0001; Figure 4, analysis 1.1.2). Meta-analysis of subgroup 3 (2 RCTs) showed that the effect of acupuncture at Tiaokou in combination with shoulder acupoints was superior to sham acupuncture at Tiaokou in combination with acupuncture at shoulder acupoints (RR = 2.47 [1.74, 3.51] (95% CI) *P* < 0.00001; Figure 4, analysis 1.1.3). Correspondingly, no statistically significant difference was observed in subgroup 4 (3 RCTs), which applied tuina, exercise of the affected shoulder, and oral NSAIDs as control interventions (RR = 1.04 [0.99, 1.10] (95% CI) *P* = 0.14; Figure 4, analysis 1.1.4).

#### 3.3.2. Evaluation on VAS

In the included RCTs, there were 10 studies [13, 15, 17, 21, 22, 25–29] which quantified the degree of shoulder pain of SAC patients using VAS.

**Figure 5:**
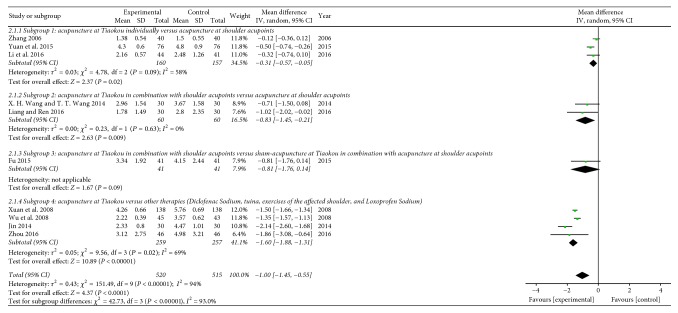
Forest plots of outcome “VAS.”

In this meta-analysis, we extracted and analyzed the data from these 10 studies and the overall acesodyne effects favored acupuncture at Tiaokou in accordance with the VAS score (WMD = −1.00 [−1.45, −0.55] (95% CI) *P* < 0.0001; Figure 5). Meanwhile, the heterogeneity test showed a high level of inconsistency across the 10 included studies (*P* < 0.00001; *I*^2^ = 94%; Figure 5). We performed a subgroup analysis to investigate the possible cause of heterogeneity. However, high levels of heterogeneity were shown in subgroup 1 and subgroup 4 (subgroup 1: *P* = 0.09, *I*^2^ = 58%; subgroup 4: *P* = 0.02, *I*^2^ = 69%); furthermore, the total subgroup differences were significantly substantial (*P* < 0.00001, *I*^2^ = 93%). We finally applied a random-effects model.

Meta-analysis of subgroup 1 (3 RCTs) showed that acupuncture at Tiaokou individually achieved a significant reduction in the VAS score, in comparison with acupuncture at shoulder acupoints (WMD = −0.31 [−0.57, −0.05] (95% CI) *P* = 0.02; Figure 5, analysis 2.1.1). Meta-analysis of subgroup 2 (2 RCTs) showed that acupuncture at Tiaokou in combination with shoulder acupoints demonstrated a more favorable effect on VAS than acupuncture at only shoulder acupoints did (WMD = −0.83 [−1.45, −0.21] (95% CI) *P* = 0.009; Figure 5, analysis 2.1.2). The meta-analysis result of subgroup 3 (1 RCT) demonstrated that there was not enough evidence to show that acupuncture at Tiaokou in combination with shoulder acupoints was superior to sham acupuncture at Tiaokou in combination with acupuncture at shoulder acupoints. (WMD = −0.81 [−1.76,0.14] (95% CI) *P* = 0.09; Figure 5, analysis 2.1.3). And in comparison with tuina, exercise of the affected shoulder, and oral NSAIDs, the meta-analysis result of subgroup 4 (4 RCTs) showed that acupuncture at Tiaokou could definitely reduce the VAS scores of SAC patients (WMD = −1.60 [−1.88, −1.31] (95% CI) *P* < 0.00001; Figure 5, analysis 2.1.4).

#### 3.3.3. Evaluation on CMS

In the included RCTs, there were four studies [22–25, 28] which quantified the recovery of shoulder mobility using CMS for SAC patients.

**Figure 6:**
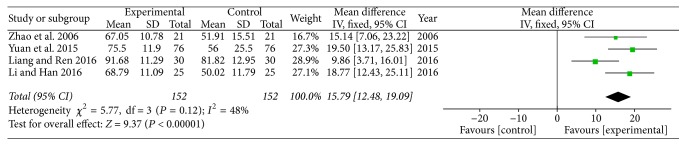
Forest plots of outcome “CMS.” Comparison: acupuncture at Tiaokou versus other therapies.

In this meta-analysis, we extracted and analyzed the data from these four studies. Meta-analysis of these four RCTs showed low heterogeneity (*P* = 0.12; *I*^2^ = 48%). We applied a fixed-effects model. The results demonstrated a favorable effect of acupuncture at Tiaokou (as the sole treatment or in combination with shoulder acupoints) on CMS (WMD = 15.79 [12.48, 19.09] (95% CI) *P* < 0.00001; Figure 6) compared with other acupuncture therapies (acupuncture at only shoulder acupoints, sham acupuncture at Tiaokou in combination with acupuncture at shoulder acupoints).

#### 3.3.4. Evaluation on Adverse Effects

None of these studies reported any severe adverse events related to acupuncture at Tiaokou. Of the concluded RCTs, safety evaluation was conducted in four trials [22, 23, 25, 26]. The incidence rate of adverse effects in one trial [23] was 6.90% as reported, with two of the participants in the experiment group experiencing subcutaneous hematoma or a painful sensation during the process. Another trial [25] reported nine participants that had felt electric shock or tingling during manipulating of needles. Meanwhile, two trials [22, 26] report the appearance of adverse effects but included no specific details. All the cases reported of adverse effects were handled with reasonable countermeasures.

#### 3.3.5. Heterogeneity Analysis

In this meta-analysis, the cause of the heterogeneity of the percentage of clinical effectiveness and VAS had been investigated using subgroup analysis. Nevertheless, heterogeneity could not readily be explained. The sources of heterogeneity may be attributed to the treatment factors. Firstly, a highly heterogeneous application of acupuncture between studies is probably a critical impediment to consistency. Different methods of needle manipulation at Tiaokou include (electroacupuncture or elongated needling method) selection of acupoints, depth of insertion, manipulation frequency, needle retention time, duration of stimuli, and parameters of electroacupuncture. Particularly, it has been verified that different electrical parameters and manipulations may show different therapeutic effects [36, 37]. Secondly, none of the included trials mentioned the practitioners' backgrounds, such as their professional affiliation, or how long they had been practicing acupuncture. Thirdly, concerning control treatments, we found that alternative control treatments had not been found except for acupuncture at shoulder acupoints, tuina, kinesiotherapy, and oral NSAIDs (Diclofenac Sodium or Loxoprofen Sodium) in included trials. The pharmaceutical drugs which are mainly used for SAC should be an important comparison for future RCT studies.

#### 3.3.6. Funnel Plot Analysis

**Figure 7:**
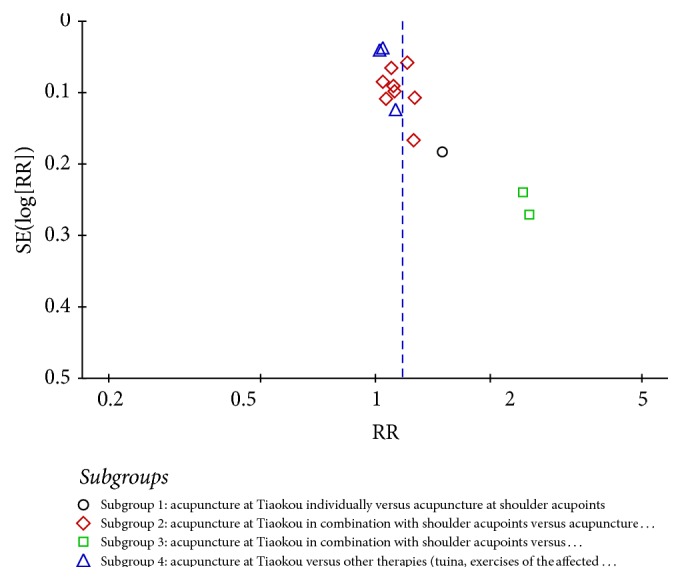
Funnel plot of outcome “percentage of clinical effectiveness.”

In the final report of this meta-analysis, there were 14 RCTs which were evaluated for the percentage of clinical effectiveness concerning acupuncture at Tiaokou in the experimental group for treating SAC. The results of publication bias analysis showed that the inverse funnel plot was asymmetrical (Figure 7).

## 4. Discussion

In TCM, the afflicted area of SAC is around the shoulder, which is comprised of the large intestine meridian of Hand Yangming and the small intestine meridian of Hand Taiyang. Generally, the pathogenesis of SAC is always due to the stagnation of Qi and blood and the obstruction of meridians around the shoulder. As an important branch of alternative therapy, acupuncture can provide a useful backup to medication or kinesiotherapy for improving the overall symptoms and minimizing side-effects. Tiaokou (ST38) belongs to the stomach meridian of Foot Yangming. From a physiological standpoint, Yangming meridian is characterized by excessive Qi and blood. To enhance the sensation of the acupuncture treatment in order to reach an outstanding curative effect, many acupuncturists always apply elongated needle therapy to penetrate from Tiaokou to Chengshan (BL57) or utilize electroacupuncture at Tiaokou for treating SAC in the clinic [38]. Chengshan belongs to the bladder meridian of Foot Taiyang, which characteristically is used to treat various pain syndromes due to lesions of muscle and tendon. Massively stimulating Tiaokou in combination with a small amount of range of motion exercises in the affected shoulder has shown to be effective in improving SAC symptoms by dredging the restriction of Qi and blood in the Yangming and Taiyang meridian [34].

### 4.1. Summary of Evidence

An irregular hierarchy of evidence quality always undermines the persuasiveness of results, which impels us to conscientiously formulate the methodology of our systematic review. Through rigorously screened 19 RCTs, we were able to present an unambiguous evaluation on the therapeutic effectiveness of acupuncture at Tiaokou for SAC. However, caution is still warranted when considering the generalizability of our results due to the inevitable bias and quality issues of the evidence.

The outcome assessment of our systematic review can be summarized in three aspects: the percentage of clinical effectiveness, VAS (assessing the relief of shoulder pain), and CMS (evaluating the recovery of shoulder joint mobility) of SAC patients. To begin with, the results of our meta-analysis revealed significant differences in acupuncture at Tiaokou (as sole treatment or in combination with shoulder acupoints) versus other acupuncture therapies (acupuncture at shoulder acupoints, sham acupuncture at Tiaokou in combination with acupuncture at shoulder acupoints) for improving the percentage of clinical effectiveness (*P* = 0.03, *P* < 0.0001, and *P* < 0.00001). But there was not enough evidence to demonstrate that acupuncture at Tiaokou could achieve a better effect than other kinds of therapy (tuina, exercise of the affected shoulder, and oral NSAIDs).

Meanwhile, the results of our meta-analysis revealed significant differences in acupuncture at Tiaokou (as sole treatment or in combination with shoulder acupoints) versus other therapies (acupuncture at shoulder acupoints, tuina, exercise of the affected shoulder, and oral NSAIDs) for the relief of shoulder pain (*P* = 0.02, *P* < 0.009, and *P* < 0.00001). What is more, compared with other therapies, acupuncture at Tiaokou appeared to reach a better therapeutic effectiveness in improving the recovery of shoulder joint mobility (*P* < 0.00001).

### 4.2. Limitations of Our Systematic Review

Despite rigorous inclusion criteria and methodology, limitations of our systematic review should be taken seriously into account. Due to the following reasons, we cannot achieve a definite conclusion about the exact curative effect of acupuncture at Tiaokou on SAC. More extensive evidence is required before this kind of treatment for SAC is widely recommended in the clinic.

#### 4.2.1. Methodological Quality of the Included Studies

From the current data outcome, the majority of the included trials were determined to be of low quality. 42.1% of the included trials (8/19) were described as RCTs simply for the reason of mentioning “randomization.” 31.6% of the included trials (6/19) reported the details and reasons for abscission. 15.8% of the included trials (3/19) reported the blinding methods. 36.8% of the included trials (7/19) reported allocation concealment. The low quality of the majority of the trials debases the strength of our systematic review. Moreover, the inverse funnel plot was asymmetrical, an exact sign of the existence of publication bias, small sample size, and low quality trials.

#### 4.2.2. Selection of the Participants of the Included Studies

The diagnostic criteria for SAC in the included studies were inconsistent. In the included RCTs, fourteen studies adopted the diagnostic criteria of SAC in* Diagnostic Criteria for Symptoms and Signs of Chinese Medicine* [33], while two studies adopted the diagnostic criteria of SAC in* Clinical Guidelines of New Drugs for Traditional Chinese Medicine* [34]. Two studies adopted the diagnosis of SAC which was formulated by the* China National Symposium on Shoulder Adhesive Capsulitis* in 1997. One study adopted the diagnosis of SAC in* Diagnostic Criteria for Clinical Diagnosis and Efficacy* [39].

All diagnostic criteria above focused on the essential symptoms of SAC, chronic shoulder pain, restriction of the shoulder joint, specific age of onset (around 50 years old), and so on. There were still several distinctions between each criterion. Selective bias, resulting from inconsistency in diagnostic criteria, may affect the outcome of our meta-analysis.

#### 4.2.3. Selection of the Needling Methods of the Included Studies

In order to dredge Qi and blood to get a better effect, all the included studies applied intense stimulus to enhance the needling sensation of Tiaokou. There are two kinds of needling methods at Tiaokou in the included trials: the elongated needling method and the electroacupuncture method. Therefore, combining data from multiple studies and needling methods set inherent limitations for our review and created difficulty in reaching a definite conclusion.

#### 4.2.4. Long-Term Effects Report of the Included Studies

In the included RCTs, only two studies [13, 27] set up follow-ups for 3 months. The lack of follow-ups made it difficult to reflect the long-term effect of acupuncture at Tiaokou for treating SAC.

#### 4.2.5. Selection of the Outcome Measures of the Included Studies

The outcome measures of the included trials were inconsistent. In several studies, VAS and CMS were used to assess the recovery of shoulder pain and restriction of the shoulder movement, but these outcomes pose difficulty in terms of worldwide acceptance. These conditions might also debase the strength of our review.

### 4.3. Implications for Future Research

In recent years, the number of clinical trials on acupuncture at Tiaokou to treating SAC has increased gradually, but the quality of studies should still be improved. Our suggestions for future research are as follows: (1) a detailed report should be presented about the random sequence generation, allocation concealment, case shedding, and withdrawal in research; (2) due to the inherent characteristics of acupuncture manipulations, it is hardly possible to conduct blinding, especially for the acupuncturists, but the blinding of participants and statistical analysis should be performed; (3) international criteria should be applied for admitting participants and evaluating therapeutic effectiveness; (4) follow-ups should be strengthened to record important clinical indicators for long-term observation.

## 5. Conclusions

On the basis of current clinical evidence, this systematic review suggests that acupuncture at Tiaokou (as sole treatment or in combination with shoulder acupoints) achieved statistically significant effects in improving the overall symptoms and the percentage of clinical effectiveness. However, the current evidence may not be sufficiently robust against potential methodological flaws and significant heterogeneity. Further large-scale, well-designed RCTs on this topic are still warranted.

## Figures and Tables

**Table 1:** Characteristics of included studies.

Study	Sample size (E/C)	Characteristics of participants:		Interventions		Course of treatment (day)	Main outcomes
		gender, ages, (E/C)					
		Experiment	Control	Experiment	Control		
Zhang 2006 [13]	40/40	17/23; 40–74	15/25; 40–68	② ④	⑤	15	PCE, VAS
Zhao et al. 2006 [14]	25/25	-/-; 35–65	-/-; 35–65	① ④ ⑤	③ ⑤	10	PCE, CMS
Xuan et al. 2008 [15]	138/138	56/82; 35–65	46/92; 35–65	② ④	⑨	5	VAS
Sun et al. 2008 [16]	78/39	32/46; 44–58	25/14; 45–60	① ④ ⑤	⑤	30	PCE
Wu et al. 2008 [17]	45/43	19/26; 40–70	18/25; 40–70	① ④ ⑤	⑧	14	PCE, VAS
Luo 2009 [18]	38/38	22/16; 44–58	25/13; 45–60	① ④ ⑤	⑤	30	PCE
Wang 2010 [19]	58/58	37/21; 44–58	39/19; 45–60	① ④ ⑤	⑤	30	PCE
Fan and Wang 2013 [20]	35/35	12/23; 42–65	9/26; 42–65	① ④ ⑤	⑤	28	PCE
X. H. Wang and T. T. Wang 2014 [21]	30/30	14/16; 48–55	13/17; 49–57	① ④ ⑤	⑤	14	PCE, VAS
Jin 2014 [22]	30/30	13/17; 35–65	14/16; 35–65	① ④	④	20	PCE, VAS
Lin 2014 [23]	29/29	9/20; 40–75	11/18; 40–75	① ④ ⑥	⑥	12	PCE
Zhang 2015 [24]	59/58	31/27; 38–68	33/25; 36–69	① ④ ⑦	⑦	30	PCE
Yuan et al. 2015 [25]	76/76	31/45; 40–75	32/44; 40–75	① ④	⑤	28	VAS, CMS
Fu 2015 [26]	41/41	25/16; 33–65	11/30; 33–65	① ④ ⑤	③ ④ ⑤	42	VAS, CMS
Liang and Ren 2016 [27]	30/30	8/22; 44–66	7/23; 45–68	① ④ ⑤	⑤	14	VAS, CMS
Li et al. 2016 [28]	44/41	16/28; 40–70	14/27; 40–70	② ④	⑥	28	VAS
Zhou 2016 [29]	46/46	-/-; 48–64	-/-; 48–64	① ④ ⑤	⑨	14	PCE, VAS
Li and Han 2016 [30]	25/25	-/-; 37–68	-/-; 37–68	① ④ ⑤	③ ④ ⑤	10	PCE
Guo 2016 [31]	128/128	68/60; 45–58	48/80; 40–59	① ④ ⑤	⑤	23	PCE

(a) Types of interventions: ① penetrating Tiaokou through Chengshan; ② electroacupuncture at Tiaokou; ③ sham-acupuncture at Tiaokou; ④ gentle range-of-motion exercises of the affected shoulder; ⑤ acupuncture at shoulder acupoints; ⑥ electroacupuncture at shoulder acupoints; ⑦ warm-acupuncture at shoulder acupoints; ⑧ tuina; ⑨ nonsteroidal anti-inflammatory drugs (Diclofenac Sodium or Loxoprofen Sodium); (b) abbreviations of main outcomes: PCE, percentage of clinical effectiveness; VAS, Visual Analogue Scale; CMS, Constant-Murley Score.

## Abbreviations

CBM: Chinese biomedicine literature
CI: Confidence intervals
CNKI: China national knowledge infrastructure
CMS: Constant-Murley Score
MD: Mean differences
NSAIDs: Nonsteroidal anti-inflammatory drugs
VAS: Visual Analogue Scale
RCTs: Randomized controlled trials
RRs: Risk ratios
SAC: Shoulder adhesive capsulitis
SMD: Standardized mean differences
TCM: Traditional Chinese medicine
WMD: Weighted mean differences.
